# Dose-Dependent Beneficial Effect of Ketone Supplement-Evoked Ketosis on Anxiety Level in Female WAG/Rij Rats: Sometimes Less Is More

**DOI:** 10.3390/nu15204412

**Published:** 2023-10-18

**Authors:** Enikő Rauch, Csilla Ari, Zsolt Kovács

**Affiliations:** 1 Department of Biology, Savaria University Centre, Eötvös Loránd University (ELTE), Károlyi Gáspár tér 4, 9700 Szombathely, Hungary; raucheniko9810@gmail.com (E.R.); zskovacsneuro@gmail.com (Z.K.); 2 Institute of Biology, University of Pécs, Ifjúság Str. 6, 7624 Pécs, Hungary; 3 Behavioral Neuroscience Research Laboratory, Department of Psychology, University of South Florida, Tampa, FL 33620, USA; 4 Ketone Technologies LLC, Tampa, FL 33612, USA

**Keywords:** exogenous ketone supplement, anxiety, LDB test, female WAG/Rij rat, mental health

## Abstract

While one-third of the population can be affected by anxiety disorders during their lifetime, our knowledge of the pathophysiology of these disorders is far from complete. Previously, it has been demonstrated in male animals that exogenous ketone supplement-evoked ketosis can decrease anxiety levels in preclinical rodent models, such as Wistar Albino Glaxo/Rijswijk (WAG/Rij) rats. Thus, in this study, we investigated whether intragastric gavage of the exogenous ketone supplement KEMCT (mix of 1,3-butanediol-acetoacetate diester/ketone ester/KE and medium-chain triglyceride/MCT oil in 1:1 ratio) for 7 days can alter the anxiety levels of female WAG/Rij rats using the light–dark box (LDB) test. We demonstrated that a lower dose of KEMCT (3 g/kg/day) increased blood R-βHB (R-β-hydroxybutyrate) levels and significantly decreased anxiety levels (e.g., increased the time spent in the light compartment) in female WAG/Rij rats on the seventh day of administration. Although the higher KEMCT dose (5 g/kg/day) increased blood R-βHB levels more effectively, compared with the lower KEMCT dose, anxiety levels did not improve significantly. We conclude that ketone supplementation might be an effective strategy to induce anxiolytic effects not only in male but also in female WAG/Rij rats. However, these results suggest that the optimal level may be moderately, not highly, elevated blood R-βHB levels when the goal is to alleviate symptoms of anxiety. More studies are needed to understand the exact mechanism of action of ketone supplementation on anxiety levels and to investigate their use in other animal models and humans for the treatment of anxiety disorders and other mental health conditions.

## 1. Introduction

It has been demonstrated that up to one-third of the population can be affected by anxiety disorders during their lifetime [1,2,3]. However, our knowledge about the mechanism of action of anxiety disorders is far from complete [1,3]. It has been demonstrated that changes, in particular GABA (gamma-aminobutyric acid)-ergic, serotonergic, glutamatergic, noradrenergic and adenosinergic systems in implicated brain areas, such as the hippocampus, prefrontal cortex and amygdala, may have a role in the pathological alterations resulting anxiety disorders [4,5,6,7]. Indeed, different drugs that can modify the above mentioned systems, such as selective serotonin reuptake inhibitors, selective serotonin–norepinephrine reuptake inhibitors and benzodiazepines, may be used to alleviate different symptoms of anxiety disorders [4,7,8]. As the efficacy of currently used anxiolytic medications is highly variable and anxiolytics may evoke several adverse side effects [8], further studies are needed to develop safer and more effective anxiolytic drugs and therapeutic approaches.

It has also been demonstrated previously that exogenous ketone supplements, such as ketone salts (KSs) and ketone esters (KEs), not only increase the blood levels of ketone bodies (ketosis) but also effectively decrease anxiety levels in different preclinical rodent models [3,9,10,11,12,13,14,15]. Moreover, the connection between the ketone supplement-evoked increase in blood ketone body β-hydroxybutyrate (βHB) levels and anxiolytic effects was also confirmed [3,9,12,14,16]. These results may suggest that ketone supplementation-generated ketosis may alleviate symptoms of anxiety disorders not only in rodent models but also in humans, and it may be a promising adjunctive treatment, but further studies are needed specifically for human applications and for the treatment of other mental health conditions [3,17,18]. However, in spite of having different ingestible ketone preparations commercially available that can effectively and safely increase blood ketone body levels, ketone therapy (e.g., oral administration of ketone supplements) in the treatment of neuropsychiatric disorders, such as anxiety, depression and bipolar disorder, has been mostly neglected [3,17,18].

While anxiolytic drugs are used in both genders in human therapy, the incidence of anxiety disorders was found to be higher in women than in men [1]. However, the ketone supplement-generated influence on anxiety disorders has been studied mainly on male preclinical animal models [9,10,11,12,13,14,15]. For example, the ketone supplementation-generated anxiolytic effect was demonstrated only by investigation of male Wistar Albino Glaxo/Rijswijk (WAG/Rij) rats [9,14]. It is important to consider that it has been demonstrated in WAG/Rij rats [19] that a ketone supplement-evoked increase in blood R-βHB levels may be gender-dependent [20] and the sex of the animals may influence the results of behavioral tests [21]. Therefore, to obtain a better understanding of the potential differences between genders, multiple types of experiments need to be performed on both genders. Thus, as a first step, in the current study, we investigated whether administration of the ketone supplement KEMCT (mix of 1,3-butanediol-acetoacetate diester/ketone ester/KE and medium-chain triglyceride/MCT oil in 1:1 ratio) for 7 days can generate changes in the anxiety levels of female WAG/Rij rats using the light–dark box (LDB) test [22,23].

## 2. Materials and Methods

### 2.1. Animals

The animals were treated according to the Hungarian Act of Animal Care and Experimentation (1998, XXVIII, section 243), European Communities Council Directive (86/609/EEC), as well as the EU Directive 2010/63/EU. The Animal Care and Experimentation Committee of the ELTE Savaria University Centre (Eötvös Loránd University) approved the experiments under license number VA/ÉBÁF-ÁO/00279-4/2021 by the National Scientific Ethical Committee on Animal Experimentation (Hungary).

Female WAG/Rij rats (n = 24; 8 months old, 171–204 g) were housed in groups (4 animals) and were kept under standard laboratory conditions. We provided free access to normal food (SSNIFF RM-Z+H rat breeding and maintenance diet, TOXI-COOP Ltd., Budapest, Hungary) and water, as well as air-conditioned room at 22 ± 2 °C. After the day of the last treatment, the rats were euthanized by isoflurane.

### 2.2. Light–Dark Box Test

The LDB test is frequently used to evaluate the anxiolytic influence of different drugs and treatments in rodent models [22,23]. The test is based on a conflict between the tendency of rodents to explore their environment and the predisposition of rodents to avoid brightly lit places [24,25]. The LDB apparatus was made from Plexiglas and consisted of two chambers. One chamber was transparent and remained uncovered during the test (light box/compartment), whereas the other chamber was black and was covered with a black lid (dark box/compartment). The two chambers were connected by a rectangular opening. The light box was illuminated by 80 lux (the light was 150 cm above the box), whereas inside the dark box, illumination was not observable (<5 lux) [23]. LDB tests were performed between 4.00 p.m. and 7.00 p.m.

During the LDB test, the animal was placed at the center of the light compartment facing away from the opening and was allowed to freely explore the apparatus for a period of 5 min. The following variables were measured: the time spent in the light compartment, the latency to exit the light compartment (time until first entry into the dark compartment), the latency to first reentry into the light compartment, the number of chamber transitions (entry was considered when all 4 paws of the rat were in the opposite chamber) and number of risk assessments (if animal looked out from the dark compartment, but it did not step into the light compartment with all 4 paws: aborted reentry into the light box) [23,25,26]. It has been demonstrated previously that decreased time spent in the light compartment, decreased latency to exit the light compartment, increased latency to first reentry into the light compartment, decreased number of chamber transitions and increased number of risk assessments are associated with an increase in anxiety-type behavior [24,25,27,28]. The behavior of the animals during the experiment was video recorded in the light compartment. Between sessions, the apparatus was cleaned using 30% ethanol, followed by water.

### 2.3. Experimental Design

We demonstrated previously that intragastric administration (gavage) of KEMCT for 7 days was well tolerated, did not evoke side effects (e.g., diarrhea) and was able not only to increase but also to maintain blood R-βHB level (ketosis) in WAG/Rij rats [29,30,31]. In this study, the KEMCT was a mix of 1,3-butanediol-acetoacetate diester (ketone ester/KE) (developed at University of South Florida, Tampa, FL, USA, and Savind Inc., Urbana, IL, USA) [32], as well as medium-chain triglyceride (MCT) oil in 1:1 ratio (containing ~60% caprylic triglyceride, ~40% capric triglyceride, Bloomingdale, IL, USA).

Animals were moved to an anteroom and were treated by gavage on all experimental days. Female WAG/Rij rats were assigned to 3 groups. In order to facilitate adapting the animals to the gavage method, we administered water by intragastric gavage (3 g/kg/day, group 1 and group 2; 5 g/kg/day, group 3) for 5 days (5 days adaptation period; group 1–group 3). Then, animals were gavaged by water (3 g/kg/day) (control, group 1, n = 8) or KEMCT (3 g/kg/day: group 2, n = 8; or 5 g/kg/day: group 3, n = 8) on 7 consecutive days. On the seventh day of water (group 1/control) or KEMCT (group 2 and group 3) gavage, LDB tests were performed one hour after gavage treatment.

To determine the effect of KEMCT on blood glucose and R-βHB levels, measurements were taken on the last (fifth) adaptation day (baseline level), as well as first and last (seventh) treatment days about 70 min after treatment (on the last/seventh day: about 5 min after LDB test; group 1–group 3). To measure blood glucose, as well as R-βHB levels, a commercially available blood glucose and ketone monitoring system (Precision Xtra, Abbott Laboratories, Abbott Park, IL, USA) was used [9,31]. For the test, blood was taken from the rats’ tail veins.

We also measured the body weight of the rats before treatments started (fifth adaptation day: baseline) and after last (seventh) treatment.

### 2.4. Statistics

Data presented are the mean ± standard error of the mean (S.E.M.). Changes in blood glucose and R-βHB levels, LDB parameters (e.g., time spent in light box) and body weight were compared to baseline (body weight), to the water-gavaged animals (control, group 1; LDB test) and to both of them (baseline and control; blood glucose and R-βHB levels). For data analysis, GraphPad Prism version 9.2.0 (using a two-way ANOVA with Tukey’s multiple comparisons test and Dunnett’s multiple comparisons test) was used. We considered the results significant when *p* < 0.05.

## 3. Results

### 3.1. Effect of KEMCT Administration on Anxiety Levels

**Figure 1:**
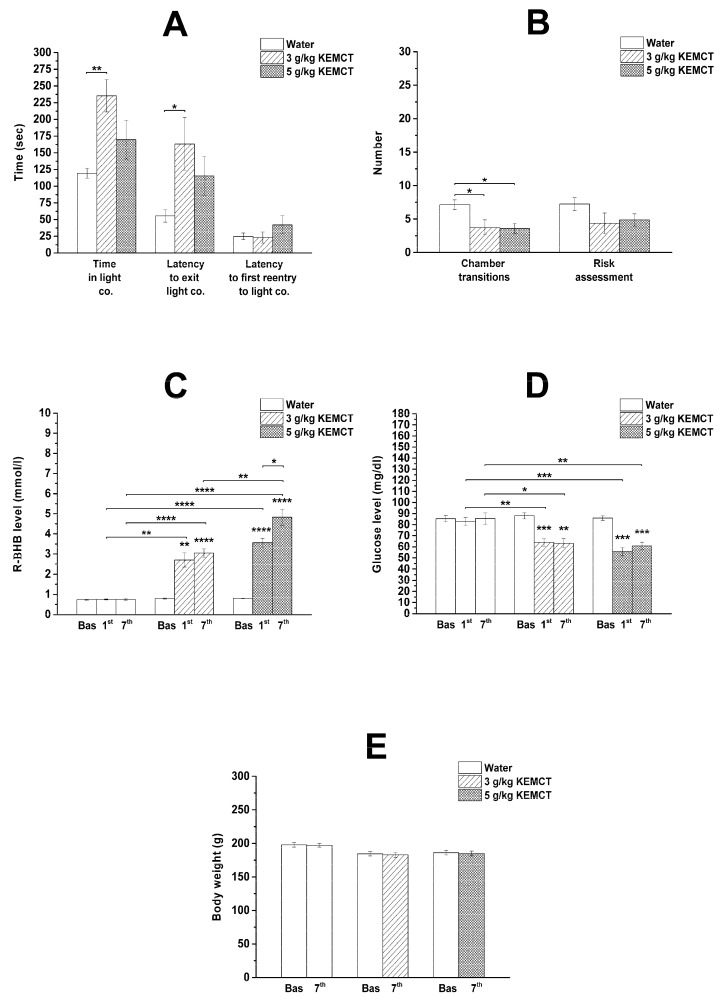
Effect of low KEMCT dose (3 g/kg/day; group 2) and high KEMCT dose (5 g/kg/day; group 3) on anxiety levels (**A**,**B**), blood R-βHB (**C**) and glucose levels (**D**), as well as body weight (**E**) compared with baseline (**E**), water-gavaged animals (control, group 1) (**A**,**B**) or both of them (baseline and control; **C**,**D**). Abbreviations: Bas, baseline; co., compartment; KEMCT, mix of ketone ester (1,3-butanediol-acetoacetate diester/KE) and medium-chain triglyceride (MCT) oil in 1:1 ratio; R-βHB, R-beta-hydroxybutyrate; * *p* < 0.05; ** *p* < 0.01; *** *p* < 0.001; **** *p* < 0.0001.

The lower dose of KEMCT (3 g/kg/day; group 2) significantly increased the time spent in the light compartment and the latency to exit the light compartment, whereas the number of chamber transitions significantly decreased on day 7 of the KEMCT treatment compared with the control (group 1) (Figure 1A,B; Table 1). In response to the high KEMCT dose (5 g/kg/day; group 3), the number of chamber transitions significantly decreased on the seventh day of KEMCT gavage (Figure 1B; Table 1). Other variables of the LDB test, such as the latency to first reentry into the light compartment and the number of risk assessments, were not changed significantly after administration of low (3 g/kg/day; group 2) or high (5 g/kg/day; group 3) KEMCT doses compared with the control (group 1) (Figure 1A,B; Table 1).

### 3.2. Effect of KEMCT Administration on Blood Levels of R-βHB and Glucose as Well as Body Weight

After administration of water gavage (control, group 1), we did not find significant differences in blood levels of R-βHB and glucose compared with baseline (Figure 1C,D; Table 2).

A significant increase in blood R-βHB levels was observed after administration of both lower (3 g/kg/day; group 2) and higher (5 g/kg/day; group 3) doses of KEMCT on the first and seventh days of the treatments compared with baseline and the control (group 1) (Figure 1C; Table 2). Moreover, gavage of a higher KEMCT dose (5 g/kg/day) was able to induce significantly higher blood R-βHB levels compared with a lower KEMCT dose (3 g/kg/day) on the seventh day of the treatment (Figure 1C). A significantly higher R-βHB level was also demonstrated after the administration of a higher KEMCT dose (5 g/kg/day) on the seventh day of administration compared with its first administration (Figure 1C).

A similar and significant decrease was demonstrated in blood glucose levels after administration of both doses of KEMCT (3 g/kg/day and 5 g/kg/day, group 2 and group 3, respectively) on the first and seventh days of the treatments compared with baseline and the control (group 1) (Figure 1D; Table 2).

A significant change in body weight was not observed after the seventh day of gavage administration of water (control, group 1) or at low (3 g/kg/day; group 2) or high (5 g/kg/day; group 3) doses of KEMCT compared with baseline (Figure 1E; Table 2).

## 4. Discussion

Following our previous study on male WAG/Rij rats, in this study performed on females, we demonstrated that administration of a lower KEMCT dose (3 g/kg/day) for 7 days not only increased blood R-βHB levels and decreased blood glucose levels but also significantly decreased some measures of anxiety levels in female WAG/Rij rats (specifically, the time spent in the light compartment and the latency to exit the light compartment increased). Although a higher KEMCT dose (5 g/kg/day) similarly decreased blood glucose levels and more effectively increased blood R-βHB levels, compared with the low KEMCT dose-generated effects, it was not effective in significantly reducing anxiety levels.

We demonstrated in female WAG/Rij rats that the time spent in the light compartment and the latency to exit the light compartment increased after administration of a lower dose of the KEMCT (3 g/kg/day) (Figure 1A). After the gavage of the higher KEMCT dose (5 g/kg/day), only the number of chamber transitions changed (decreased) significantly. However, a non-significant increase was observed, not only in the time spent in the light compartment and the latency to exit the light compartment, but also in the latency to first reentry into the light compartment after the gavage of the higher KEMCT dose. Moreover, there was a non-significant decrease in risk assessments after the administration of both KEMCT doses. Thus, these results suggest that the administration of a lower KEMCT dose may be able to induce anxiolytic effects, but a higher KEMCT dose did not decrease anxiety levels effectively (Figure 1A,B). Nevertheless, the number of chamber transitions decreased after the gavage of the lower KEMCT dose (3 g/kg/day) (Figure 1B), suggesting a discrepancy with the anxiolytic influence of the lower KEMCT dose described above. We speculated that this controversial result may be in association with the KEMCT-generated high increase in both the time spent in the light compartment and the latency time to exit the light compartment compared with the results of the control group (group 1) (Figure 1A). Namely, based on the data in Figure 1A and Table 1, the percentages of the session time spent in the light compartment for the control group (group 1) and KEMCT-treated group (3 g/kg/day, group 2) were 39.8 and 78.5, respectively. Moreover, the latency to exit the light compartment was 2.95 times longer after the gavage of 3 g/kg of KEMCT compared with the control group (theoretically, more time spent in the light compartment, less time and moderate tendency to enter the dark compartment and, as a consequence, chamber transition). However, in relation to the LDB test, it was demonstrated that the most reliable and consistent sign of decreased anxiety is the increased time spent in the light compartment [24,25]. Thus, as the time spent in the light compartment significantly increased in group 2, we could conclude that the administration of a lower KEMCT dose (3 g/kg/day) had an anxiolytic effect in female WAG/Rij rats.

In spite of that, a ketone supplement-evoked increase in blood βHB levels may correlate with a ketone supplement-generated anxiolytic effect [3,9,12,14,16], and the higher KEMCT dose (5 g/kg/day) enhanced more effectively the blood levels of R-βHB compared with the lower KEMCT dose-generated effects, only the gavage of the lower dose of KEMCT (3 g/kg/day) increased the time spent in the light compartment significantly (Figure 1A,C). As there was no significant improvement in the time spent in the light compartment after administration of the higher KEMCT dose, these results suggest that generating higher R-βHB levels by administration of a higher KEMCT dose might not be the most effective approach to induce anxiolytic effect in females. Moreover, it has been demonstrated previously that KEMCT (2.5 g/kg/day, gavage for 7 days) generated higher R-βHB levels than the same dose of KEKS (mix of KE and KS). In spite of this last result, KEKS increased the time more effectively until immobility in isoflurane-anesthetized WAG/Rij rats compared with KEMCT [30], while KEKS and KEMCT similarly increased the latency to fall on an accelerating rotarod test in WAG/Rij rats [33]. All of these results suggest that a proper range of R-βHB levels may be needed for different applications and to alleviate symptoms of different diseases, such as anxiety, and to improve physiological functions, and higher doses might not always work most effectively. Therefore, we conclude that inducing very high levels of blood R-βHB may not always be necessary and optimal to enhance the efficacy of treatment for various applications.

We did not find a correlation between the decrease in blood glucose concentration in response to different doses and anxiety levels in rats. However, we demonstrated a similar decrease in blood glucose levels after administration of both doses of KEMCT (3 g/kg/day and 5 g/kg/day) for 7 days (Figure 1D). Given that only the lower KEMCT dose (3 g/kg/day) had an anxiolytic effect (Figure 1A,B) and the administration of another ketone supplement KSMCT (mix of KS and MCT oil) did not decrease blood glucose concentrations significantly but significantly mitigated anxiety levels on the seventh treatment day in male rats [14], we hypothesized that decreased blood glucose levels likely do not have the main role in the KEMCT-evoked alleviating influence on anxiety levels. It has been demonstrated previously that widespread neuronal death occurs only when blood glucose levels decrease below 1 mM (~18 mg/dL) in both mice and rats [34], while neuroprotective effects in very low glucose level situations may be connected to the astrocyte–neuron lactate shuttle [35,36,37]. Given that after the administration of the KEMCT, the lowest glucose level was 55.75 ± 3.54 mg/dL in our study (Table 2), we suggest that the effect of KEMCT on anxiety levels likely was not associated with hypoglycemia-evoked cell (neuronal) damage.

It has been demonstrated that following oral consumption or intragastric gavage, ketone esters, such as 1,3-butanediol-acetoacetate diester, are entirely hydrolyzed in the small intestine by esterases, and 1,3-butanediol is converted to acetoacetate and βHB in the liver by alcohol and aldehyde dehydrogenase [38,39,40]. Moreover, MCTs can be hydrolyzed to medium-chain fatty acids by lipases in the gastrointestinal tract, which are also converted to ketone bodies in the liver [41]. Finally, ketone bodies, such as βHB, can exit the liver, be transported to the central nervous system through the bloodstream and be utilized for ATP (adenosine triphosphate) synthesis in neurons [42,43]. In relation to the mechanism of action of KEMCT on anxiety levels, it was suggested that enhanced levels of ketone bodies, such as βHB, may increase the level of adenosine in the central nervous system [44]. Indeed, it has been demonstrated that ketone bodies enhance not only the intracellular concentration of ATP in neurons but also the neuronal release of ATP, as well as the extracellular level of adenosine by the ectonucleotidases-generated catabolism of ATP [42]. Moreover, previous in vivo and in vitro studies show in rodents that after the digestion of ketone supplements [38,39,40], increased levels of blood βHB may be able to enhance adenosine concentration in different brain areas, such as the striatum [44,45,46,47]. Increased neuronal levels of adenosine can enhance the activation of A1-type adenosine receptors (A1Rs), which may open ATP-sensitive potassium (K_ATP_) channels and generate neuronal hyperpolarization of central nervous system neurons, resulting in a decrease in excitatory neurotransmitter release [42,48,49,50]. Accordingly, it was demonstrated that βHB can decrease glutamate release and neuronal excitability [51], likely via enhanced activity of A1Rs. This influence may evoke an anxiolytic effect through, for example, attenuation of glutamate-evoked hyperexcitability of brain areas (e.g., prefrontal cortex) implicated in the emergence of anxiety disorders [7,52,53]. Thus, ketone supplement-induced ketosis could alleviate levels of anxiety, likely through enhanced activity of neuronal A1Rs [5,42,54,55]. These results suggest that similarly to the KSMCT-generated ketosis in male WAG/Rij rats [14], the KEMCT-evoked increase in R-βHB levels in the present study may also generate an anxiolytic effect via the adenosinergic system in female WAG/Rij rats. Low basal extracellular levels of adenosine [56] and likely a slight increase in adenosine levels preferentially activate neuronal A1Rs, resulting in inhibitory effects in the central nervous system, such as a decrease in glutamate release [57]. Nevertheless, a higher increase in adenosine levels can activate A2A-type adenosine receptors (A2ARs) in the A1R–A2AR heteromer, blocking the functions of A1Rs and generating enhanced glutamate release [57,58]. Thus, it is possible that administration of higher doses of ketone supplements (e.g., 5 g/kg KEMCT/day) could generate higher levels of not only blood R-βHB (Figure 1C) but also adenosine compared with the effects evoked by lower doses of ketone supplements, in which higher adenosine levels may be enough for the preferential activation of neuronal A2ARs [59]. Increased activation of A2ARs may enhance glutamatergic neurotransmission [57], and glutamate-evoked hyperexcitability in the implicated brain areas of anxiety disorders may result in a mitigated (abolished) anxiolytic effect compared with lower doses of KEMCT-generated alleviating influence (Figure 1A,B), likely through A1Rs.

Administration of KEMCT, theoretically, may modulate not only the adenosinergic and glutamatergic but also other neurotransmitter systems (e.g., GABAergic and serotonergic system) of different brain areas through, for example, increased ketone body levels, resulting in a decrease in anxiety [7,60,61]. Moreover, R-βHB may also exert its effects on functions and diseases of the central nervous system through G-protein coupled hydroxycarboxylic acid receptor 2 (HCAR2 or GPR109 receptor) and free fatty acid receptor 3 (FFAR3) [43,62]. However, further studies are needed to understand the exact role of neurotransmitter systems, receptors and other factors (e.g., other ketone bodies, such as acetoacetate, which were not measured in this study) by which ketone supplements, such as KEMCT, are able to generate an anxiolytic effect.

There are several limitations of the current study. Those limitations include that we investigated the KEMCT-evoked effect on anxiety levels by using only one age group (8 months old) of female WAG/Rij rats, and only one method, the LDB test, was used. However, the current study aimed to further validate and extend our previous studies on the effects of ketone supplementation on anxiety levels in similarly aged male WAG/Rij rats [9,14]. While the LDB test is a commonly used method for the investigation of anxiety levels in rats [22,63,64], and the LDB test is frequently used alone to investigate anxiety-like behavior [25,28,65], additional studies using other age groups and methods will be needed to further evaluate the current results. It also needs to be considered that the results of the behavioral testing may be affected by the strain and estrous cycle of animals, as well as the drug delivery method [21], and these potential influencing factors were not considered in this study. These influences will need to be taken into consideration in future studies (e.g., using other strains and behavioral tests, such as the elevated plus maze/EPM test) in order to increase confidence in the presented results and to obtain a more comprehensive understanding of the influence of ketone supplementation on anxiety levels. Moreover, further studies are needed to find the most effective formulations and the optimal doses of ketone supplements to mitigate anxiety levels, to investigate the effect of ketone supplementation-evoked increases in not only βHB levels but also acetoacetate levels in relation to ketosis-evoked anxiolytic influences. It will be important to extend the studies to reveal the effect of chronic treatment using ketone supplements on anxiety levels, as well as to measure how long the anxiolytic influence would last in response to chronic treatment. The current study did not measure changes in other neurotransmitter systems in response to ketone supplementation, which will also be important to better understand the exact mechanisms of action.

## 5. Conclusions

The current results extended our previous studies performed on male WAG/Rij rats by using the LDB test on female WAG/Rij rats. Therefore, we can conclude that ketone supplementation may evoke an anxiolytic effect not only in male, but also in female WAG/Rij rats. Our results also show that there may be a dose-dependent influence of ketone supplementation on central nervous system diseases, such as anxiety. We found that the alleviating effect of ketosis on anxiety levels cannot necessarily be increased by higher levels of blood ketone bodies. Consequently, we conclude that inducing an optimal range of blood R-βHB (ketosis) levels may be needed for the effective treatment of anxiety disorders, and high levels might not be optimal, at least in females, for this application. These results strengthen our hypothesis that ketone supplement-evoked nutritional ketosis may be a useful therapeutic tool to alleviate symptoms of anxiety disorders; however, more detailed studies are needed for specific applications, for example, for diabetic or therapy-resistant human patients or for other mental health conditions.

## Figures and Tables

**Table 1:** Effect of KEMCT (3 g/kg/day and 5 g/kg/day; group 2 and group 3, respectively) administration for 7 days on anxiety levels in female WAG/Rij rats compared with water-gavaged animals (control, group 1). Abbreviations: KEMCT, mix of ketone ester (1,3-butanediol-acetoacetate diester/KE) and medium-chain triglyceride (MCT) oil in 1:1 ratio; ns, non-significant; * *p* < 0.05; ** *p* < 0.01.

	Water (Control; Group 1; Mean ± S.E.M.)	3 g/kg KEMCT (Group 2; Mean ± S.E.M.; Level of Significance/*p*-Value)	5 g/kg KEMCT (Group 3; Mean ± S.E.M.; Level of Significance/*p*-Value)
Time spent in light compartment (s)	119.4 ± 7.34 -	235.5 ± 24.09 **/0.0025	169.8 ± 29.0 ns/0.2106
Latency to exit light compartment (s)	55.4 ± 9.19 -	163.3 ± 39.43 */0.0271	115.5 ± 28.75 ns/0.2582
Latency to first reentry to the light compartment (s)	24.8 ± 4.89 -	23.0 ± 8.29 ns/0.9877	42.0 ± 13.43 ns/0.3527
Number of chamber transitions	7.1 ± 0.72 -	3.8 ± 1.13 */0.0351	3.6 ± 0.75 */0.0284
Number of risk assessments	7.3 ± 0.99 -	4.4 ± 1.55 ns/0.2234	4.9 ± 0.92 ns/0.3509

**Table 2:** Influence of water (control, group 1) and KEMCT (3 g/kg/day and 5 g/kg/day; group 2 and group 3, respectively) administration on blood R-βHB and glucose levels as well as body weight in female WAG/Rij rats compared with baseline. Abbreviations: KEMCT, mix of ketone ester (1,3-butanediol-acetoacetate diester/KE) and medium-chain triglyceride (MCT) oil in 1:1 ratio; ns, non-significant; R-βHB, R-beta-hydroxybutyrate; ** *p* < 0.01; *** *p* < 0.001; **** *p* < 0.0001.

	Blood R-βHB Level (mmol/L; Mean ± S.E.M.; Level of Significance/*p*-Value)	Blood Glucose Level (mg/dL; Mean ± S.E.M.; Level of Significance/*p*-Value)	Body Weight (g; Mean ± S.E.M.; Level of Significance/*p*-Value)
**Group 1 (Control)**			
Baseline	0.74 ± 0.04 -	85.25 ± 2.71 -	197.75 ± 3.31 -
First gavage	0.75 ± 0.03 ns/0.9329	82.88 ± 3.32 ns/0.5448	- -
Seventh gavage	0.75 ± 0.03 ns/0.9719	85.50 ± 5.18 ns/0.9979	197.25 ± 2.72 ns/0.9587
**Group 2 (3 g/kg KEMCT)**			
Baseline	0.80 ± 0.03 -	88.00 ± 2.87 -	184.25 ± 3.03 -
First gavage	2.70 ± 0.36 **/0.0027	64.00 ± 3.54 ***/0.0002	- -
Seventh gavage	3.05 ± 0.21 ****/<0.0001	63.25 ± 4.00 **/0.0057	182.75 ± 3.59 ns/0.4633
**Group 3 (5 g/kg KEMCT)**			
Baseline	0.81 ± 0.03 -	85.88 ± 1.99 -	186.00 ± 3.38 -
First gavage	3.56 ± 0.22 ****/<0.0001	55.75 ± 3.54 ***/0.0002	- -
Seventh gavage	4.83 ± 0.40 ****/<0.0001	60.88 ± 3.28 ***/0.0003	184.75 ± 3.58 ns/0.6092

## Data Availability

The data presented in this study are available on request from the corresponding author.

## Abbreviations

A1R, A1-type adenosine receptors; A2AR, A2A-type adenosine receptors; ATP, adenosine triphosphate; βHB, beta-hydroxybutyrate; GABA, gamma-aminobutyric acid; KE, ketone ester; KEKS, a mix of KE and KS; KEMCT, a mix of KE and MCT oil; KS, ketone salt; KSMCT, a mix of KS and MCT oil; LDB, light–dark box; MCT, medium-chain triglyceride; WAG/Rij, Wistar Albino Glaxo/Rijswijk
